# Addiction as a cardiometabolic disease: a neurocardiometabolic framework and the emerging role of GLP-1 receptor therapies

**DOI:** 10.1093/ehjopen/oeag101

**Published:** 2026-07-03

**Authors:** Jose Seijas-Amigo, Ángel Salgado-Barreira, Sonia Eiras, Susana Arias-Rivas, Gemma Rodriguez-Carnero, Moisés Rodriguez-Mañero, Jose Ramon Gonzalez-Juanatey

**Affiliations:** Cardiology Department, Complejo Hospitalario Universidad de Santiago de Compostela, Travesía da Choupana s/n, Santiago de Compostela 15706, Spain; Fundación Instituto de Investigación Sanitaria de Santiago de Compostela (FIDIS), Santiago de Compostela 15706, Spain; Centro de Investigación Biomédica en Red de Enfermedades Cardiovasculares (CIBERCV), Madrid 28029, Spain; University of Santiago de Compostela, Santiago de Compostela 15782, Spain; University of Santiago de Compostela, Santiago de Compostela 15782, Spain; Department of Preventive Medicine and Public Health, University of Santiago de Compostela, Santiago de Compostela 15782, Spain; Consortium for Biomedical Research in Epidemiology and Public Health (CIBER en Epidemiología y Salud Pública CIBERESP), Carlos III Health Institute, Madrid 28029, Spain; Centro de Investigación Biomédica en Red de Enfermedades Cardiovasculares (CIBERCV), Madrid 28029, Spain; Traslational Cardiology Group, Health Research Institute, Santiago de Compostela 15706, Spain; Neurology Department, Complejo Hospitalario Universidad de Santiago de Compostela, Santiago de Compostela 15706, Spain; Endocrinology Department, Complejo Hospitalario Universidad de Santiago de Compostela, Santiago de Compostela 15706, Spain; Cardiology Department, Complejo Hospitalario Universidad de Santiago de Compostela, Travesía da Choupana s/n, Santiago de Compostela 15706, Spain; Cardiology Department, Complejo Hospitalario Universidad de Santiago de Compostela, Travesía da Choupana s/n, Santiago de Compostela 15706, Spain; Fundación Instituto de Investigación Sanitaria de Santiago de Compostela (FIDIS), Santiago de Compostela 15706, Spain

**Keywords:** GLP-1 receptor agonists, Cardiometabolic disease, Substance-use disorders, Addiction medicine, Cardiovascular prevention

## Abstract

Substance-use disorders are major accelerators of cardiometabolic disease, yet this dimension remains insufficiently recognized within addiction care. Individuals with substance-use disorders frequently exhibit hypertension, adverse lipid profiles, visceral adiposity, metabolic syndrome, chronic low-grade inflammation and autonomic imbalance, contributing to markedly elevated cardiovascular morbidity and premature mortality. The recent European Society of Cardiology Clinical Consensus Statement on mental health and cardiovascular disease highlights this gap and calls for structured cardiometabolic assessment and prevention strategies within behavioural and psychiatric population. Glucagon-like peptide-1 receptor therapies have emerged as multisystem agents relevant to both addictive behaviour and cardiometabolic health. Experimental and early clinical evidence demonstrates reductions in craving, improved reward regulation and attenuation of impulsive consumption across alcohol, nicotine, stimulant use and binge-type eating, mediated through gut–brain communication, mesolimbic dopamine circuitry, hypothalamic pathways and stress-responsive networks. In parallel, these therapies induce clinically meaningful reductions in body weight, visceral adiposity, blood pressure and inflammatory burden, while improving glucose regulation and cardiometabolic markers. Large cardiovascular outcome trials demonstrate reductions in major adverse cardiovascular events in high-risk populations, and emerging primary-prevention analyses report favourable changes in estimated cardiovascular risk even without established cardiovascular disease. This review integrates biological, clinical and real-world evidence to propose a unified neurocardiometabolic framework in which glucagon-like peptide-1 receptor therapies may simultaneously influence addictive behaviour and long-term cardiovascular risk trajectories. We outline mechanistic foundations and potential clinical applications within detoxification programmes, relapse-prevention settings, dual-diagnosis services and addiction care, while highlighting the need for further clinical validation before routine implementation.

## Graphical Abstract

**Graphical Abstract:**
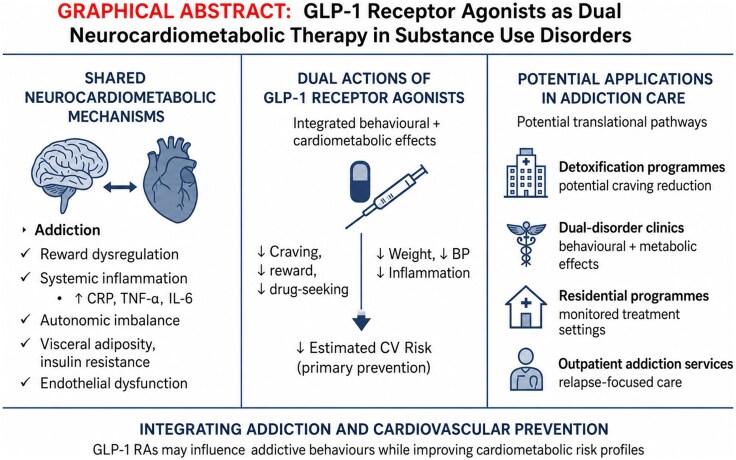

## Introduction

Substance-use disorders (SUDs) are among the most powerful accelerators of cardiometabolic disease, yet this dimension remains largely under-recognized in addiction medicine.^1,2^ Far beyond their psychiatric classification, SUDs generate a systemic phenotype characterized by chronic low-grade inflammation, autonomic dysregulation, impaired glucose–insulin homeostasis, and enhanced oxidative stress—hallmarks shared with obesity, diabetes, and premature atherosclerosis.^3–5^ Individuals with SUDs exhibit strikingly elevated rates of hypertension, dyslipidaemia, central obesity, and metabolic syndrome, often surpassing those observed in general cardiology populations.^6,7^ Notably, cardiovascular disease constitutes the leading cause of death in alcohol use disorder and a major contributor to excess mortality in stimulant and opioid use disorders, frequently exceeding deaths due to overdose.^8–10^

This clinical gap has been explicitly highlighted in the recently published 2025 ESC Clinical Consensus Statement on mental health and cardiovascular disease,^11^ which identifies the intersection between mental health, addiction, and cardiovascular disease as one of the major unmet needs in preventive cardiology. The document calls for the integration of cardiometabolic risk assessment, early prevention strategies, and collaborative care pathways within mental-health and addiction settings—representing a paradigm shift in how cardiovascular risk should be conceptualized and managed in individuals with SUDs. Our review directly responds to this call, expanding the framework proposed by the ESC by incorporating the emerging role of GLP-1 receptor agonists as dual neurometabolic agents.

In parallel, glucagon-like peptide-1 receptor agonists (GLP-1 RAs) have evolved from glucose-lowering agents to multisystem neurometabolic therapies. Preclinical and early clinical studies demonstrate that GLP-1 signalling modulates reward processing, craving and impulsivity across several addictive behaviours—including alcohol, nicotine, cocaine and binge-type eating—via coordinated actions on mesolimbic dopamine pathways, hypothalamic regulation and gut–brain–reward circuits.^12–16^ These findings support the growing hypothesis that GLP-1 RAs may directly influence addiction-related neurobiology.

Concurrently, robust evidence has established the cardiovascular safety and efficacy of GLP-1 receptor agonists across a broad spectrum of cardiometabolic risk. Large cardiovascular outcome trials, obesity-focused studies, and contemporary meta-analyses consistently demonstrate reductions in major adverse cardiovascular events, cardiovascular mortality, and cardiometabolic risk markers in individuals with type 2 diabetes, obesity, and high cardiovascular risk, with emerging evidence also supporting benefit in primary-prevention settings.^17–27^ More recent studies with oral GLP-1 receptor agonists and obesity-focused trials further extend these findings beyond injectable formulations and beyond glycaemic control alone.^23,24,26,28^ Real-world observational cohorts and prospective primary-prevention analyses additionally suggest favourable effects on estimated long-term cardiovascular risk trajectories in individuals without established cardiovascular disease.^29–32^

The convergence of addiction neurobiology and cardiometabolic medicine creates a compelling therapeutic opportunity. Patients with SUDs represent one of the highest cardiovascular-risk groups encountered in clinical practice, yet structured cardiovascular risk screening and prevention remain uncommon within addiction services.^33,34^ Integrating GLP-1 receptor agonists into detoxification, relapse-prevention, and dual-disorder programmes could therefore redefine addiction treatment, shifting the focus from short-term substance-related outcomes towards long-term cardiovascular risk modification.

In this review, we propose a unified neurocardiometabolic framework in which GLP-1 RAs act as dual-mechanism therapies targeting both addictive behaviour and cardiovascular risk. We synthesize the evidence supporting this hypothesis, outline potential clinical integration pathways, and present the clinical implications of this therapeutic model.

## Methods

This article is a narrative and integrative review that synthesizes evidence across preclinical, translational, clinical, and real-world domains to develop a unified neurocardiometabolic framework linking SUDs and cardiovascular disease through GLP-1 signalling.

Rather than aiming to perform an exhaustive systematic review or meta-analysis, we sought to critically integrate representative and high-impact evidence spanning addiction neuroscience, cardiometabolic physiology, and cardiovascular prevention, in line with the conceptual and hypothesis-generating nature of this work.

A structured literature search was conducted in PubMed/MEDLINE, Embase, Scopus, and Web of Science, covering publications from January 2000 to January 2026. Search terms combined keywords and controlled vocabulary related to:

- GLP-1 receptor agonists and incretin-based therapies;
- SUDs and compulsive behaviours; and
- cardiometabolic and cardiovascular outcomes, including primary prevention.

In addition, manual searches of reference lists from key articles, major reviews, landmark cardiovascular outcome trials, and relevant translational or mechanistic studies were performed to ensure inclusion of seminal and contemporary evidence.

The structured search identified 742 records, of which 511 remained after duplicate removal. After screening titles and abstracts, 164 full-text articles were assessed, and 102 studies were considered relevant for inclusion in the final narrative synthesis. A structured search flowchart is provided in Supplementary material online, *Figure S1*.

Evidence was selected based on biological plausibility, methodological quality, clinical relevance, and contribution to the proposed framework, rather than on rigid inclusion or exclusion criteria. The review therefore incorporates a broad spectrum of study designs, including preclinical models, human laboratory studies, randomized controlled trials, real-world observational cohorts, and mechanistic physiological investigations.

Where appropriate, the overall methodological robustness of evidence across domains was considered using established quality frameworks adapted to the study type. This assessment was used to contextualize findings and identify knowledge gaps, rather than to exclude studies from the synthesis.

## The neurocardiometabolic hypothesis: a dual-mechanism role of GLP-1 receptor agonists

The convergence of addiction biology and cardiometabolic dysregulation suggests that a single therapeutic strategy may be capable of modifying both domains. We propose that GLP-1 RAs represent the first pharmacological class with credible potential to simultaneously modulate addictive behaviour and reduce long-term cardiovascular risk in individuals with SUDs. This hypothesis is grounded in three complementary observations.

First, GLP-1 signalling directly modulates reward circuitry. Experimental studies demonstrate that central GLP-1 receptors in the ventral tegmental area (VTA), nucleus accumbens, and lateral septum regulate dopamine release, attenuate drug-induced reinforcement, and reduce motivated drug-seeking behaviour.^35–38^ Across various substances—including alcohol, nicotine, stimulants and binge-type eating—GLP-1 agonism reduces craving, cue reactivity, and compulsive intake in both preclinical and early human studies.^39–43^ These effects are consistent with a broader role of GLP-1 in regulating impulsivity, stress responsivity, and executive control.^44^

Second, GLP-1 RAs exert potent cardiometabolic actions that counteract the systemic phenotype highly prevalent in SUD populations. These agents induce substantial weight loss, improve insulin sensitivity, lower blood pressure, reduce systemic inflammation, and favourably modify lipid metabolism—all mechanisms implicated in premature atherosclerosis and cardiovascular mortality among patients with SUDs.^45–47^ Cardiovascular outcome trials have demonstrated reductions in major adverse cardiovascular events in high-risk populations with established cardiovascular disease,^17–26^ while emerging evidence from primary-prevention analyses confirms improvements in cardiometabolic markers and estimated cardiovascular risk even in individuals without known cardiovascular disease.^29–32^

Third, the pathophysiological overlap between addiction and cardiometabolic disease provides a mechanistic substrate for dual-domain benefit. Both conditions share dysregulation of the hypothalamic–pituitary–adrenal axis, chronic sympathetic activation, impaired endothelial function, microglial activation, and metabolic inflammation—biological networks that GLP-1 signalling is known to influence.^45–48^ This shared neuroimmune–metabolic architecture supports the concept that GLP-1 RAs may alter the long-term trajectory of cardiometabolic disease in SUDs while also modulating short-term addictive behaviour.

Taken together, these lines of evidence support a unified neurocardiometabolic hypothesis:

- GLP-1 receptor agonists may act as dual-mechanism therapeutics capable of reducing substance-related behaviours and simultaneously modifying cardiovascular risk trajectories in high-risk individuals.

If validated, this paradigm would reposition addiction treatment as an opportunity for early cardiovascular prevention—an urgent shift given the disproportionately high cardiometabolic burden and premature mortality seen in SUD populations.

**Figure 1:**
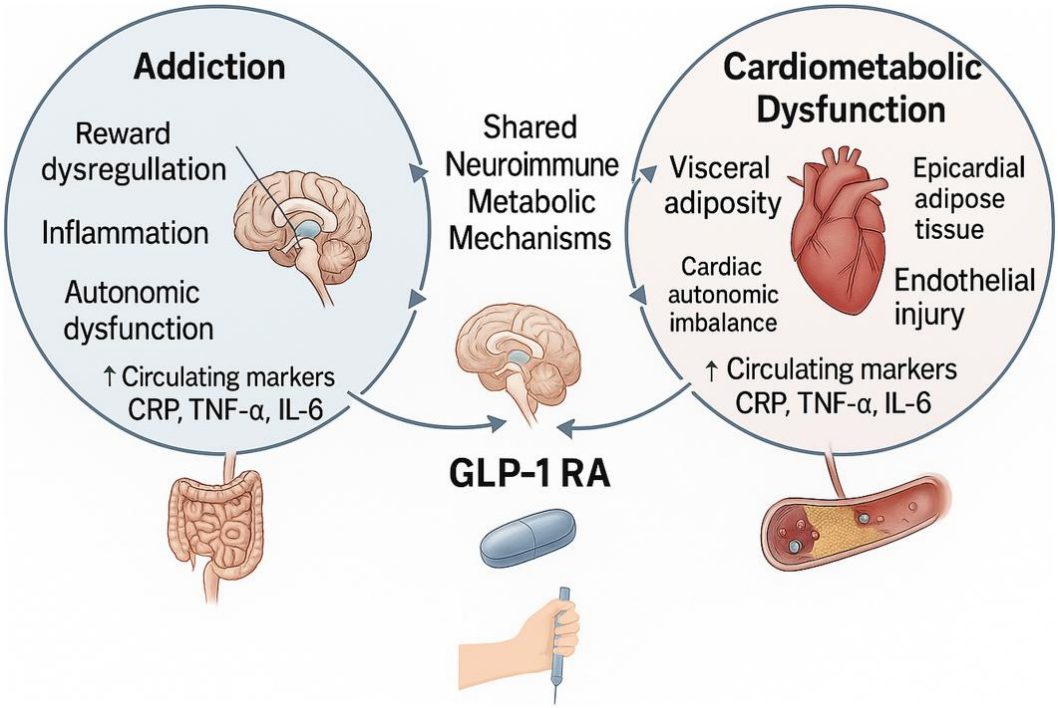
Shared neurocardiometabolic mechanisms linking addiction and cardiometabolic disease. Addiction and cardiometabolic dysfunction appear to share convergent pathways, including reward-circuit dysregulation, systemic inflammation, autonomic imbalance, visceral adiposity with insulin resistance, and endothelial injury. GLP-1 receptor agonists may modulate several of these interconnected neuroimmune–metabolic systems.

This unified neurocardiometabolic model, integrating shared mechanisms and dual-domain effects of GLP-1 RAs, is summarized in the *Figure 1*.

## Biological rationale: shared mechanisms linking addiction and cardiometabolic disease

Addiction and cardiometabolic disease, traditionally viewed as separate clinical entities, share a deep and underappreciated overlap in neurobiological, inflammatory, and metabolic pathways. This mechanistic convergence provides the conceptual foundation for a dual-domain therapeutic approach using GLP-1 RAs.

### Gut–brain–reward signalling as a common pathway

SUDs disrupt gut-derived metabolic signals—including GLP-1, ghrelin, PYY, and leptin—which play essential roles in reward modulation and homeostatic feeding.^45,49–55^ Chronic substance exposure alters enteroendocrine secretion, vagal afferent signalling, and hypothalamic regulation, resulting in heightened reward sensitivity and impaired satiety pathways.^55^ GLP-1 RAs restore these signals, attenuating drug-induced dopamine release within the ventral tegmental area and nucleus accumbens, reducing cue reactivity and decreasing compulsive drug seeking.^35–38,56–58^ The same gut–brain circuits regulate glucose metabolism, appetite control, and cardiovascular autonomic tone, underpinning the systemic reach of GLP-1 biology.^59^

### Mesolimbic dopamine and executive-control networks

Addictive substances dysregulate dopamine signalling, promoting sensitization, motivational salience and loss of inhibitory control.^60^ Parallel impairments in prefrontal cortex activity lead to heightened impulsivity and reduced behavioural regulation—a profile also observed in obesity, binge eating, and metabolic syndrome.^61,62^ GLP-1 RAs modulate both subcortical reward pathways and cortical executive networks, reducing impulsivity and improving cognitive control in preclinical models and emerging human data.^63–65^ These effects provide a plausible mechanism for multi-substance benefit.

### Systemic and neuroinflammation as a shared driver

Both addiction and cardiometabolic disease involve chronic low-grade inflammation, characterized by elevated IL-6, TNF-α, CRP, and activation of microglia and peripheral immune cells.^66–68^ Alcohol, cocaine, nicotine, and opioids all trigger inflammatory cascades that accelerate endothelial dysfunction, insulin resistance, and vascular injury.^69–71^ GLP-1 RAs exert anti-inflammatory effects in peripheral tissues and the central nervous system, reducing cytokine expression, attenuating oxidative stress, and modulating microglial activation.^72–74^ This anti-inflammatory profile is relevant for both reducing relapse vulnerability and limiting long-term cardiovascular damage.

### Autonomic dysregulation and stress-system activation

SUDs produce chronic sympathetic overdrive, impaired baroreflex sensitivity, dysregulated hypothalamic–pituitary–adrenal (HPA) axis activity, and increased cardiovascular reactivity to stress—core contributors to hypertension, arrhythmias, and adverse cardiovascular outcomes.^4,48,66,69–71^ Similar patterns of autonomic imbalance occur in obesity, diabetes, and sleep-related metabolic disorders.^44,45,47^ GLP-1 signalling influences autonomic output, decreasing sympathetic activity, improving vagal tone, and normalizing HPA-axis responsiveness in experimental models.^44,45,47,59,72^ These autonomic effects align with both short-term craving reduction and longer-term cardiometabolic benefit.^11–15,38–42,56–58,72,73^

### Metabolic dysfunction and endothelial injury

Individuals with SUDs exhibit high rates of visceral adiposity, insulin resistance, dyslipidaemia, and metabolic inflexibility—even in the absence of obesity.^6,7,66,71^ These factors accelerate endothelial dysfunction, arterial stiffness, and subclinical atherosclerosis, contributing to premature cardiovascular events. GLP-1 RAs improve insulin sensitivity, reduce visceral fat, enhance endothelial nitric oxide bioavailability, and decrease markers of vascular inflammation and oxidative stress.^19,45–47,72^ These mechanisms contribute to the well-documented reductions in cardiometabolic risk and may be particularly relevant for SUD populations, who experience metabolic deterioration early in life.

Weight loss likely represents an important mediator of the cardiometabolic benefits observed with GLP-1 receptor agonists, including reductions in blood pressure, systemic inflammation, visceral adiposity, and estimated cardiovascular risk. However, several cardiovascular outcome trials and mechanistic studies suggest that part of the observed benefit may extend beyond weight reduction alone, potentially involving direct effects on endothelial function, autonomic regulation, inflammatory signalling, and neurohormonal pathways.^75,76^ Nevertheless, disentangling weight-dependent from weight-independent mechanisms remains challenging, particularly in addiction-related populations. Importantly, most currently available human data derive from individuals with obesity, overweight, type 2 diabetes, or elevated cardiometabolic risk, whereas normoweight populations remain underrepresented in existing studies. Therefore, extrapolation of these findings to normoweight individuals with SUDs should be approached cautiously.

### Neuroimmune–metabolic cross-talk as the unifying model

Taken together, addiction and cardiometabolic disease share a common pathophysiological architecture involving intertwined reward, inflammatory, autonomic, and metabolic systems. GLP-1 RAs act across each of these layers, providing a biologically coherent explanation for their potential dual action. See *Figure 1*.

## Clinical evidence across two domains: addiction and cardiometabolic outcomes

GLP-1 RAs occupy a unique position in modern therapeutics: they possess robust clinical evidence in cardiometabolic medicine and rapidly growing evidence in addiction neuroscience. Although originally developed for glycaemic control, GLP-1 RAs now demonstrate integrated behavioural and physiological effects relevant across multiple addictive disorders. This section summarizes the clinical data supporting their dual-domain relevance.

### Evidence in addictive behaviours

#### Alcohol use disorder

Alcohol represents the substance with the most advanced level of clinical evidence. Extensive preclinical studies consistently show that GLP-1 receptor agonism reduces ethanol intake and suppresses conditioned alcohol-seeking behaviour in rodent models.^11,40,57,58^

These findings are supported by human laboratory studies demonstrating reduced alcohol self-administration, attenuated cue-induced craving, and diminished reward valuation under GLP-1 RA treatment.^11,12,38,56–58^

Importantly, early randomized clinical trials further extend these observations, indicating that GLP-1 RAs, including semaglutide and exenatide, reduce alcohol intake, heavy drinking days, and craving intensity in individuals with alcohol use disorder (AUD).^12,38–41^

More recently, randomized controlled evidence has strengthened this signal. In a 26-week, double-blind, placebo-controlled trial in treatment-seeking patients with alcohol use disorder and comorbid obesity, once-weekly semaglutide significantly reduced heavy drinking days compared with placebo, alongside improvements in secondary alcohol-related outcomes and a favourable safety profile.^77^ These findings provide some of the strongest clinical evidence currently available supporting a therapeutic effect of GLP-1 receptor agonists in AUD, while still requiring confirmation in larger and more diverse populations.

#### Nicotine and tobacco use disorder

In nicotine dependence, preclinical evidence demonstrates that GLP-1 receptor agonists reduce nicotine intake and attenuate relapse-like behaviour in rodent models.^14,43^

Emerging human data, including observational studies and early clinical reports, suggest reductions in cigarette consumption and craving during GLP-1 RA therapy in individuals treated for obesity or diabetes.^43,56,57^

Preliminary randomized evidence remains limited but indicates potential attenuation of smoking reward and improvements in abstinence-related markers.^43,65^ Overall, these findings support biological plausibility, although robust clinical confirmation is still lacking.

#### Cocaine and stimulant use disorders

For cocaine and stimulant use disorders, the evidence base remains predominantly preclinical. Experimental studies consistently show that GLP-1 receptor agonism attenuates cocaine-seeking behaviour, reduces drug-induced dopamine release, and disrupts relapse-related pathways.^15,36,56,69,71^

Human data are currently scarce, with no large randomized clinical trials available. Early translational observations and real-world reports suggest potential reductions in stimulant use among individuals receiving GLP-1 RAs for metabolic indications, but these findings remain exploratory and require formal validation.^58,73^

#### Binge-type eating and compulsive behaviours

Outside alcohol, the strongest clinical signal is observed in binge-type eating and reward-driven overeating. Multiple randomized controlled trials and cohort studies have shown that GLP-1 RAs, including liraglutide, dulaglutide, and semaglutide, significantly reduce binge-eating episodes, compulsive food intake, and reward-driven hunger.^63,78–80^

These clinical findings are supported by mechanistic and preclinical data demonstrating modulation of central reward pathways and feeding behaviour, reinforcing the role of GLP-1 signalling in shared neurobiological circuits underlying addictive and compulsive phenotypes.

Synthesis across addictive behaviours:

Taken together, the available evidence suggests that GLP-1 receptor agonists exert consistent effects across multiple addictive behaviours, including alcohol consumption, nicotine use, stimulant reward, and compulsive eating. However, the strength and maturity of evidence vary substantially across domains.

Randomized clinical data are currently most robust in alcohol use disorder and binge-type eating, whereas nicotine and stimulant use disorders remain largely supported by preclinical and early human evidence. As summarized in *Table 1*, this heterogeneity highlights both the therapeutic potential of GLP-1–based interventions and the need for well-designed clinical trials to establish their efficacy across different SUDs.

**Table 1:** Effects of GLP-1 receptor agonists on substance use and compulsive behaviours

Substance/behaviour	Type of evidence	Main addiction-related findings	GLP-1 RAs studied	Maturity level
Alcohol	Preclinical + phase 2 RCTs + human laboratory studies + observational evidence	↓ intake, ↓ heavy drinking days, ↓ craving, ↓ reward valuation; reduced self-administration	Semaglutide, liraglutide, exenatide	Most advanced; replicated signal across preclinical and early clinical studies
Nicotine/tobacco	Preclinical + pilot human trials + observational	↓ cigarette consumption, ↓ craving, ↓ smoking reward, ↓ reinstatement	Exenatide, dulaglutide	Promising; early-stage
Cocaine and stimulants	Preclinical	↓ drug reward, ↓ reinstatement, ↓ drug-induced dopamine release	Exendin-4, liraglutide	Strong preclinical evidence; human trials needed
Binge eating/compulsive eating	Multiple RCTs	↓ binge episodes, ↓ compulsive drive, ↓ reward-based eating	Semaglutide, liraglutide	Most consistent clinical evidence outside AUD
Other compulsive or impulsive behaviours (preclinical)	Preclinical	↓ impulsivity, ↓ compulsive seeking (preclinical models)	Exendin-4	Exploratory

Summary of preclinical and clinical evidence on GLP-1 receptor agonists across substance-use and compulsive behaviours. Alcohol use disorder and binge-eating disorder show the strongest and most consistent clinical signals, while nicotine and stimulant effects are supported mainly by preclinical and early human data.

Abbreviations: GLP-1 RA, Glucagon-like peptide-1 receptor agonist; RCT, Randomized controlled trial

Large-scale observational data further support a potential multi-substance effect. A recent target trial emulation study reported that initiation of GLP-1 receptor agonists was associated with reduced risks of incident SUDs and related adverse outcomes across multiple substances.^81^ These findings extend the hypothesis of a broad behavioural effect but remain observational and require confirmation in randomized clinical trials. Despite encouraging mechanistic and early clinical findings, substantial translational gaps remain across several substance-use domains. While alcohol use disorder currently has the most mature randomized evidence, many other indications continue to rely predominantly on preclinical, observational, or exploratory human studies, underscoring the need for adequately powered clinical trials.

A structured overview of the current clinical and translational evidence is provided in *Table 2*.

**Table 2:** Clinical and translational evidence of GLP-1 receptor agonists in addiction and related mental health conditions

Indication	GLP-1 RA	Key study	Design	Population	Main outcome	Stage of evidence
Alcohol use disorder	Semaglutide	Klausen et al., 2026	Phase 2 RCT	AUD + obesity	↓ heavy drinking days	Positive phase 2 evidence
Alcohol use disorder	Exenatide	Klausen et al., 2022	RCT	AUD	No effect on primary endpoint; ↓ cue reactivity	Early clinical evidence
Alcohol use disorder	Semaglutide	Hendershot et al., 2025	Phase 2 RCT	AUD	↓ alcohol self-administration, ↓ craving	Phase 2 clinical evidence
Multiple SUDs	GLP-1 RAs	Cai et al., 2026	Observational study	T2D veterans	↓ incident SUDs, ↓ adverse outcomes	Real-world evidence
Nicotine dependence	Exenatide	Yammine et al., 2021	Pilot RCT	smokers	↑ abstinence probability, ↓ craving	Early clinical evidence
Nicotine dependence	Dulaglutide	Lengsfeld et al., 2023	RCT	Smokers	No effect on abstinence; ↓ post-cessation weight gain	Early clinical evidence
Cocaine use disorder	Exenatide	Angarita et al., 2021	Pilot RCT	Cocaine use disorder	No acute effect	Exploratory clinical evidence
Binge eating disorder	Semaglutide/liraglutide	Multiple RCTs	RCTs	Established clinical evidence/obesity	↓ binge episodes ↓ compulsive eating	Established clinical evidence

Abbreviations: AUD, alcohol use disorder; BED, binge-eating disorder; GLP-1 RA, glucagon-like peptide-1 receptor agonist; RCT, randomized controlled trial; SUD, substance-use disorder; T2D, type 2 diabetes.

### Evidence in cardiometabolic and cardiovascular outcomes

#### Metabolic improvement in high-risk populations

GLP-1 RAs produce substantial weight loss, improve glycaemic control, reduce visceral adiposity, and favourably modify blood pressure and lipid profiles across diverse populations.^44–47^ These pleiotropic metabolic effects directly counter cardiometabolic risk factors that are highly prevalent in individuals with SUDs.

#### Cardiovascular outcome trials (CVOTs)

Large cardiovascular outcome trials have consistently demonstrated reductions in major adverse cardiovascular events (MACE), body weight, and cardiometabolic risk markers across patients with type 2 diabetes, obesity, and high cardiovascular risk (*Table 3*).^16–26^

**Table 3:** Randomized, real-world, and summary evidence supporting cardiometabolic and cardiovascular benefits of GLP-1 receptor agonists (GLP-1 RAs)

Study/evidence	Population	GLP-1 RA	Main cardiovascular findings
ELIXA	T2D post–acute coronary syndrome	Lixisenatide	Neutral on MACE (CV safety)
LEADER	T2D + established CVD	Liraglutide	↓ MACE; ↓ CV mortality
SUSTAIN-6	T2D, very high CV risk	Semaglutide (SC)	↓ MACE
EXSCEL	T2D	Exenatide ER	Neutral on MACE
REWIND	T2D; many without prior CVD	Dulaglutide	↓ MACE (primary prevention signal)
HARMONY Outcomes	T2D + ASCVD	Albiglutide	↓ MACE
AMPLITUDE-O	T2D + high CV risk	Efpeglenatide	↓ MACE
PIONEER-6	T2D + high CV risk	Oral semaglutide	CV safety; nominal signal on CV death
SOUL	T2D + high CV risk	Oral semaglutide	↓ MACE
SELECT	Overweight/obesity without diabetes	Semaglutide (SC)	↓ MACE
Meta-analysis of GLP-1 RA CVOTs (Galli et al., JACC 2025)	Mixed (T2D ± obesity; incl. primary prevention)	Multiple	↓ MACE and CV mortality; consistent benefit across molecules
SURMOUNT-5 post-hoc analysis	Obesity without diabetes or established CVD	Tirzepatide vs. semaglutide	↓ predicted 10-year CV risk (greater with tirzepatide)
SEVERAL Study (real-world, primary prevention)	Primary prevention in T2D	Multiple	↓ estimated 10-year CV risk (ASCVD/SCORE)
Mechanistic cardiology trials (HFpEF, CAD, post-MI)	Selected cardiovascular populations	Multiple	Improved endothelial function; ↓ inflammation; favourable haemodynamic effects

This table summarizes key human studies evaluating glucagon-like peptide-1 receptor agonists across substance-use disorders and related behavioural conditions. Evidence includes randomized controlled trials, early-phase interventional studies, and large-scale observational analyses. Alcohol use disorder currently has the most robust clinical evidence, including recent randomized trial data, whereas other substance domains remain supported primarily by early clinical or observational findings. The table highlights the heterogeneity in study design, populations, and outcomes, as well as the translational gap between preclinical promise and clinical validation. Observational findings should be interpreted cautiously and do not establish causality.

Abbreviations: ASCVD, atherosclerotic cardiovascular disease; CV, cardiovascular; CVOT, cardiovascular outcome trial; HFpEF, heart failure with preserved ejection fraction; MACE, major adverse cardiovascular events; SC, subcutaneous; T2D, type 2 diabetes

More recently, cardiovascular and metabolic outcome programmes involving oral GLP-1 receptor agonists have extended these findings beyond injectable formulations, reinforcing the potential cardiometabolic benefits of incretin-based therapies across different routes of administration.^23,24,82,83^

#### Meta-analysis evidence in non-diabetic and mixed populations

Contemporary meta-analyses integrating CVOTs and large randomized trials—including populations without diabetes—have confirmed that GLP-1 RAs reduce MACE and cardiovascular mortality across diverse cardiometabolic risk strata.^27^ These findings suggest that the cardiovascular benefits of GLP-1 RAs extend beyond glucose lowering and are mediated, at least in part, through improvements in endothelial function, systemic inflammation, autonomic regulation, and energy balance.

#### Evidence in primary prevention: subanalyses and real-world cohorts

Emerging evidence indicates that cardiovascular risk reduction with GLP-1 receptor agonists may occur before overt cardiovascular disease develops. Beyond traditional CVOTs, post-hoc and dedicated primary-prevention analyses have demonstrated significant improvements in cardiometabolic risk profiles and predicted cardiovascular risk among individuals without established cardiovascular disease.

In particular, a recent post-hoc analysis of the SURMOUNT-5 trial showed that, in people with obesity without type 2 diabetes or prior CVD, treatment with tirzepatide and semaglutide was associated with significant reductions in estimated 10-year cardiovascular risk, with a greater absolute risk reduction observed with tirzepatide.^28^ These findings support the concept that incretin-based therapies may confer meaningful cardiovascular benefit upstream of clinical disease onset.

Consistent with these results, contemporary meta-analyses integrating randomized trials across diabetic and non-diabetic populations confirm reductions in major adverse cardiovascular events and cardiovascular mortality with GLP-1 receptor agonists, reinforcing the plausibility of benefit in primary-prevention settings.^27^

Real-world evidence further supports this paradigm. Prospective observational cohorts, including our own SEVERAL Study, have demonstrated significant reductions in predicted 10-year cardiovascular risk (ASCVD/SCORE) among individuals without prior cardiovascular disease treated with GLP-1 receptor agonists.^32^

#### Evidence from cardiology-specific trials

Beyond diabetes and obesity, GLP-1 RAs have been investigated in cardiology-specific settings, including coronary artery disease, heart failure with preserved ejection fraction, post–acute myocardial infarction, and atrial fibrillation.^84–87^ Although clinical outcomes vary across populations, these trials consistently report favourable effects on endothelial function, inflammatory pathways, myocardial energetics, autonomic tone, and exercise capacity—mechanistic domains that overlap substantially with the cardiometabolic dysregulation observed in SUDs (*Table 3*).

### Synthesis: clinical plausibility of dual benefit

The available evidence across addiction-related and cardiometabolic domains supports the clinical plausibility of a unified neurocardiometabolic framework. Experimental and early clinical studies suggest that GLP-1 receptor agonists may modulate addictive behaviours across multiple substances by attenuating reward sensitivity, craving, and impulsivity, while cardiovascular outcome trials and real-world studies demonstrate favourable effects on cardiometabolic health and cardiovascular risk profiles, including in primary-prevention settings. Individuals with SUDs frequently lie at the intersection of these processes, exhibiting both marked cardiometabolic vulnerability and dysregulated reward-related neurobiology. Taken together, these findings provide a biologically coherent rationale for further evaluating GLP-1 receptor agonists as potential dual-mechanism therapies targeting both addictive behaviours and long-term cardiovascular risk in high-risk populations.

## Integrating GLP-1 RAs into addiction medicine: clinical pathways and implementation models

Despite the compelling overlap between addiction neurobiology and cardiometabolic disease, addiction treatment systems rarely incorporate structured cardiovascular prevention or disease-modifying therapies. GLP-1 RAs, with their dual behavioural and cardiometabolic actions, may represent a novel opportunity to explore integrated addiction and cardiometabolic care using a unified neurocardiometabolic framework. This section outlines practical pathways for integrating GLP-1 RAs into clinical addiction settings.

### Rationale for early integration in detoxification and relapse-prevention programmes

Detoxification settings represent a critical period in which neurobiological vulnerability, metabolic instability and cardiovascular stress peak.^88,89^ Early GLP-1 RA initiation may offer several advantages:

- Reduction in craving and cue reactivity during the high-risk early abstinence window.^38–42^
- Improvement in autonomic balance and stress responsivity, mitigating relapse triggers.^38–42^.^90,91^
- Rapid cardiometabolic improvements—weight, glucose, blood pressure—that directly counter the elevated cardiovascular risk seen in SUD populations.^44–47^
- Opportunity for longitudinal follow-up and titration within structured programmes.

Future integration of GLP-1 RAs during detoxification may help extend addiction treatment approaches beyond short-term withdrawal management toward broader long-term cardiometabolic risk reduction strategies.

### GLP-1 RAs as adjuncts in dual-disorder and psychiatric comorbidity services

Patients with SUDs and psychiatric comorbidities (e.g. depression, bipolar disorder, anxiety, PTSD) exhibit disproportionate rates of metabolic syndrome, obesity and cardiovascular morbidity.^92–94^ GLP-1 RAs may uniquely benefit this group through:

- Modulation of reward and stress circuits implicated in both addiction and mood disorders.^59,93^
- Improvement in energy balance, sleep, inflammation and metabolic health—all determinants of psychiatric prognosis.^66–68^
- Potential reduction in psychotropic-induced weight gain, particularly relevant for antipsychotic-treated individuals.^95,96^

Dual-disorder clinics may represent suitable environments for future evaluation and implementation studies.

### Integration into residential programmes (Minnesota model, therapeutic communities, 12-step–based care)

Residential programmes traditionally focus on psychological rehabilitation and peer-based support but rarely include structured medical cardiometabolic management.^97^ GLP-1 RA integration within these settings could:

- Provide weekly supervised dosing, ensuring adherence and reducing misuse risk.
- Address binge-type behaviours and compulsive eating, common in early abstinence.^78–80^
- Facilitate cardiovascular risk screening and early preventive therapy—components absent in most residential models but urgently needed given the premature cardiovascular mortality in SUD populations.^8–10^

These settings may provide structured environments in which GLP-1-based interventions could be prospectively evaluated alongside behavioural approaches.

### Embedding GLP-1 RAs into outpatient addiction clinics and opioid substitution therapy

Outpatient addiction services (including methadone, buprenorphine and extended-release naltrexone programmes) offer a high-frequency contact structure ideal for monitoring GLP-1 RA treatment:

- Weekly or monthly GLP-1 RA administration synchronizes with routine follow-up.
- Co-administered with opioid substitution therapy, GLP-1 RAs may reduce overlapping metabolic/inflammatory burden.^69–71^
- Opportunity for cardiovascular risk profiling (BP, lipids, glucose, atherosclerotic cardiovascular disease ASCVD/SCORE2 risk).
- Capacity to implement relapse-prevention counselling around improved craving control.

Such integration may provide an opportunity to evaluate addiction clinics as potential platforms for cardiovascular prevention in high-risk populations.

### Practical considerations: safety, dosing, monitoring and comorbidities

GLP-1 RAs are well tolerated and safe in diverse populations,^17–27^ but addiction settings require attention to:

- GI side effects during early abstinence, mitigable through slow titration.^98^
- Malnutrition or low BMI in some opioid/stimulant users—requiring careful assessment.^95^
- Hepatic dysfunction in alcohol use disorder; most GLP-1 RAs remain safe, but baseline evaluation is needed.^16^
- Drug interactions: negligible with most psychotropics and opioid agonists.^46^
- Adherence: weekly or monthly formulations improve feasibility vs. daily medications.

These considerations may support the feasibility of future implementation studies across SUD treatment environments.

### Addiction care as a potential platform for cardiovascular prevention

Integrating GLP-1 RAs into addiction treatment may support a broader conceptual framework in which SUD care also incorporates cardiovascular risk reduction strategies beyond substance-related outcomes:

- Targeting the shared pathology underlying addiction and cardiometabolic disease.
- Addressing the leading cause of mortality in SUD populations (cardiovascular disease).
- Leveraging addiction services—high-contact, longitudinal, interdisciplinary—to implement preventive cardiometabolic therapy earlier than in standard primary care.
- Potentially reducing healthcare burden through fewer future cardiovascular events, complications and relapses.

**Figure 2:**
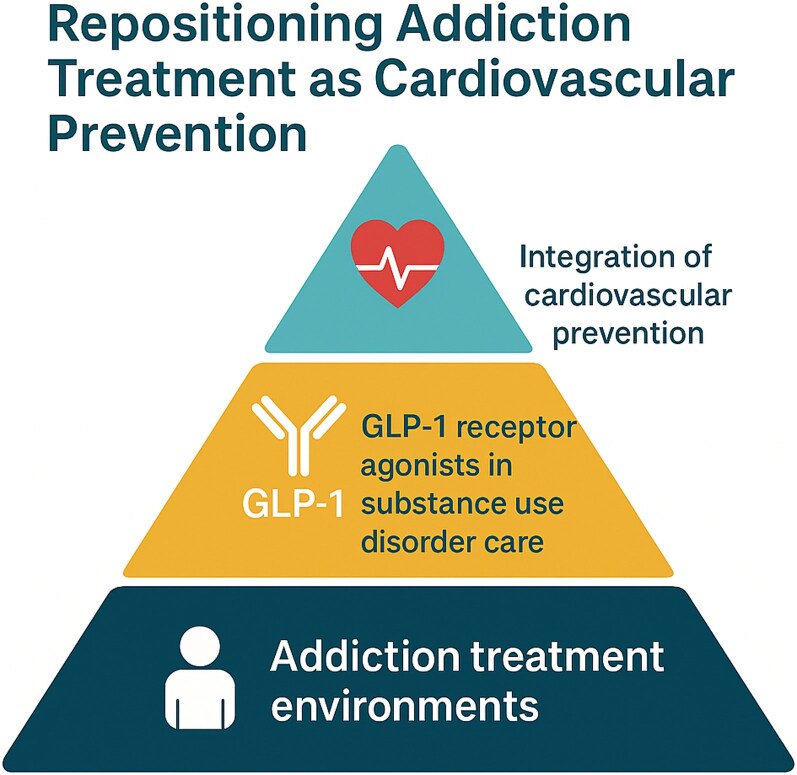
Conceptual framework: repositioning addiction treatment as cardiovascular prevention. Addiction treatment settings may represent a potential framework for evaluating GLP-1 receptor agonists as dual-behavioural and cardiometabolic interventions, within the aim of integrating cardiovascular prevention strategies into addiction care pathways.

Crucially, this approach directly aligns with the priorities of the 2025 ESC Clinical Consensus Statement on Mental Health and Cardiovascular Disease,^11^ which calls for structured cardiometabolic assessment, cardiovascular risk modification, and integration of preventive cardiology strategies within mental health and addiction pathways. The proposed neurocardiometabolic framework is conceptually aligned with these ESC recommendations and supports further investigation of addiction services as potential settings for integrated cardiovascular prevention strategies. See *Figure 2*.

## Clinical implications: redefining addiction treatment as cardiovascular prevention

The convergence between addiction neurobiology and cardiometabolic disease presents a potentially important clinical and research opportunity. Integrating GLP-1 RAs into addiction medicine may help expand how clinicians conceptualize and approach SUDs. Several practical implications emerge.

### Addiction treatment as a point of entry for cardiovascular prevention

Patients with SUDs exhibit some of the highest cardiometabolic risk profiles in medicine.^8–10^ Addiction clinics—unlike primary care—maintain frequent, structured contact during vulnerable periods such as detoxification and early abstinence. This may make them suitable environments for:

- systematic cardiovascular risk screening (blood pressure, lipids, glucose, liver function, ASCVD/SCORE2 risk)^28,32^
- early initiation of cardiometabolic therapies
- behavioural support aligned with pharmacotherapy
- coordinated follow-up with primary care and cardiology

Positioning addiction care within a cardiometabolic prevention framework may facilitate earlier intervention than traditional cardiovascular pathways.

Importantly, this strategy is conceptually aligned with several recommendations from the 2025 ESC Clinical Consensus Statement on Mental Health and Cardiovascular Disease,^11^ which emphasizes systematic cardiometabolic assessment and proactive cardiovascular risk management within mental health and addiction services.

### GLP-1 RAs as adjuncts in integrated dual-disorder care

Comorbid psychiatric disorders compound both relapse vulnerability and cardiovascular risk.^99–102^ GLP-1 RAs may offer:

- attenuation of impulsivity, emotional dysregulation and reward hypersensitivity^35,44^
- mitigation of psychotropic-induced weight gain^95,96^
- improvements in sleep, appetite regulation and inflammatory burden
- reduction of cardiometabolic risk factors common in severe mental illness

These potential effects may be relevant within multidisciplinary dual-disorder clinics.

### Potential synergy with behavioural approaches and psychosocial rehabilitation

GLP-1 RAs do not replace psychosocial interventions but may **enhance their effectiveness** by reducing craving, stress responsivity and reward dysregulation. Integration with:

- CBT and relapse-prevention therapy
- 12-step facilitation
- Minnesota-model residential programmes
- contingency management
- motivational interviewing

may potentially improve adherence, retention and long-term outcomes.^103,104^

### Implications for cardiovascular risk assessment in addiction care

For cardiology, the neurocardiometabolic model emphasizes that SUDs are associated with substantial cardiovascular risk and may merit more proactive cardiometabolic assessment. Cardiologists may consider to:

- screen routinely for alcohol, nicotine, stimulants and compulsive behaviours
- refer high-risk patients to addiction services early
- consider GLP-1 RAs when cardiometabolic risk is elevated
- integrate addiction-focused questions into secondary prevention clinics

This approach is conceptually consistent with contemporary cardio-metabolic care frameworks.

### Implementation feasibility and health-system impact

The safety profile, weekly dosing schedules and metabolic benefits of GLP-1 RAs may support future implementation studies across addiction services:

- fewer relapse-driven medical visits
- reduced progression to diabetes, hypertension and metabolic syndrome
- decreased long-term cardiovascular events
- improved quality of life and functional recovery

Given the substantial burden of SUD-related cardiovascular morbidity and mortality, the potential public health implications warrant further investigation. Emerging evidence suggests that GLP-1 receptor agonists may represent a promising neurocardiometabolic strategy to help bridge the gap between addiction medicine and cardiovascular prevention highlighted in the 2025 ESC^11^ consensus statement on mental health and cardiovascular disease.

## Conclusion

SUDs may be conceptualized as accelerated cardiometabolic conditions driven by overlapping neurobiological, inflammatory, autonomic, and metabolic mechanisms. GLP-1 receptor agonists appear to act at this intersection, with emerging evidence suggesting potential effects on reward-related behaviours alongside improvements in cardiometabolic health. Findings from neuroscience, cardiovascular outcome trials, and early clinical and observational studies support the hypothesis of a unified neurocardiometabolic framework.

GLP-1 receptor agonists may represent a promising pharmacological class with the potential to influence both addictive behaviours and long-term cardiovascular risk in vulnerable populations. Their future integration into detoxification, relapse-prevention, dual-diagnosis, and residential care settings warrants further investigation and clinical validation before routine implementation.

This conceptual framework aligns with the growing recognition highlighted in the 2025 ESC Clinical Consensus Statement on mental health and cardiovascular disease that cardiometabolic prevention strategies should be more closely integrated into behavioural and addiction-related care pathways.

## Supplementary Material

oeag101_Supplementary_Data

## Data Availability

No new datasets were generated or analysed for this review.
