# Supplementary material for: Addiction as a cardiometabolic disease: a neurocardiometabolic framework and the emerging role of GLP-1 receptor therapies
Source: Eur Heart J Open. 2026 Jul 3;6(4):oeag101. doi: 10.1093/ehjopen/oeag101 (PMC13329405; doi:10.1093/ehjopen/oeag101)
Supplement: oeag101_Supplementary_Data [file oeag101_supplementary_data.zip › Table S1.docx]

**Table S1. Quality appraisal of evidence across experimental, clinical and real-world domains**

| **Domain** | **Study Types Included** | **Appraisal framework** | **Quality Rating** | **Comments** |
| --- | --- | --- | --- | --- |
| **Preclinical (rodent/animal models)** | Behavioural assays; neurochemical studies; GLP-1 modulation of reward pathways | ARRIVE criteria (adapted) | Moderate–High | Consistent mechanistic findings; limitations include species differences. |
| **Translational neuroscience** | Human laboratory studies; neuroimaging; cue-reactivity paradigms | NIH Study Quality Assessment Tool | Moderate | Strong mechanistic plausibility; sample sizes typically small. |
| **Randomized controlled trials (addiction)** | AUD trials; early nicotine and binge-eating trials | Cochrane Risk of Bias 2.0 | Moderate | RCTs available mainly for AUD and binge eating; need larger, adequately powered trials. |
| **Randomized controlled trials (cardiovascular/metabolic)** | LEADER, SUSTAIN-6, REWIND, AMPLITUDE-O, SOUL | Cochrane Risk of Bias 2.0 | High | Large, well-conducted multicentre RCTs with robust cardiovascular endpoints. |
| **Observational real-world studies** | Cardiometabolic primary-prevention cohorts; SUD cohorts on GLP-1 | ROBINS-I | Moderate | Good external validity; limitations include residual confounding. |
| **Systematic reviews / meta-analyses** | Reviews of GLP-1 in metabolism, cardiovascular risk, and addiction | AMSTAR-2 | Variable (Moderate–High) | Strong evidence base for cardiometabolic outcomes; smaller and emerging evidence base in addiction. |
| **Mechanistic physiology / autonomic studies** | Gut–brain signalling; autonomic tone; inflammation | NIH Tool for Non-randomized Studies | Moderate | Mechanistically coherent; heterogeneous methodologies across studies. |

**Table S1.** Quality assessment of all evidence included in the review across preclinical, translational, clinical, observational and mechanistic domains. Overall evidence ratings were qualitatively assigned using validated tools appropriate to each study type, including ARRIVE for animal research, NIH Quality Assessment Tools for human laboratory and mechanistic studies, Cochrane Risk of Bias 2.0 for randomized controlled trials, ROBINS-I for observational studies, and AMSTAR-2 for systematic reviews and meta-analyses. The table summarises overall methodological quality, key strengths, and domain-specific limitations relevant to interpretation of the evidence.

**Abbreviations:**

ARRIVE – Animal Research: Reporting of In Vivo Experiments;
AMSTAR-2 – *A Measurement Tool to Assess Systematic Reviews 2*;
AUD – Alcohol Use Disorder;
CV – Cardiovascular;
GLP-1 – Glucagon-like peptide-1;
NIH – National Institutes of Health;
RCT – Randomized Controlled Trial;
RoB 2.0 – Cochrane Risk of Bias Tool 2.0;
ROBINS-I – Risk Of Bias In Non-randomized Studies of Interventions;
SUD – Substance Use Disorder.
