# Supplementary figures and images for: Addiction as a cardiometabolic disease: a neurocardiometabolic framework and the emerging role of GLP-1 receptor therapies
Source: Eur Heart J Open. 2026 Jul 3;6(4):oeag101. doi: 10.1093/ehjopen/oeag101 (PMC13329405; doi:10.1093/ehjopen/oeag101)

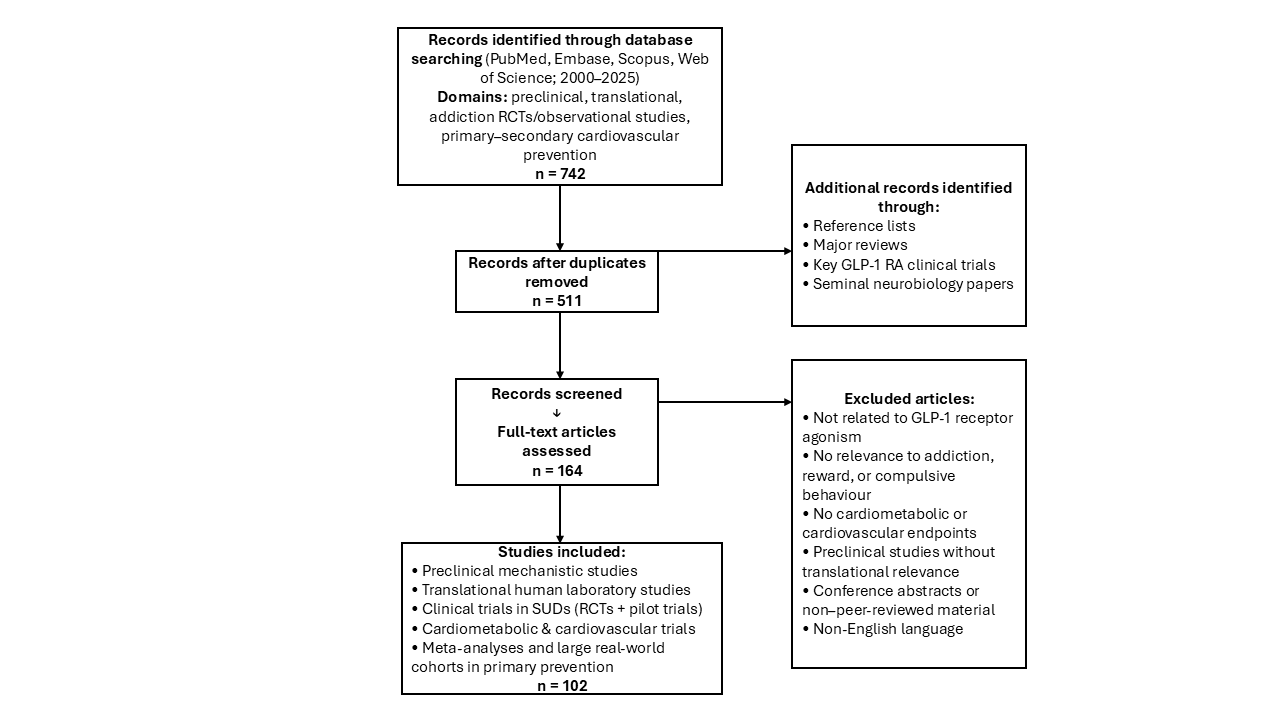

Supplement: oeag101_Supplementary_Data [file oeag101_supplementary_data.zip › Figure S1 v2.png]
